# Novel Random Copolymers of Poly(butylene 1,4-cyclohexane dicarboxylate) with Outstanding Barrier Properties for Green and Sustainable Packaging: Content and Length of Aliphatic Side Chains as Efficient Tools to Tailor the Material’s Final Performance

**DOI:** 10.3390/polym10080866

**Published:** 2018-08-04

**Authors:** Giulia Guidotti, Michelina Soccio, Valentina Siracusa, Massimo Gazzano, Andrea Munari, Nadia Lotti

**Affiliations:** 1 Civil, Chemical, Environmental and Materials Engineering Department, University of Bologna, Via Terracini 28, 40131 Bologna, Italy; giulia.guidotti9@unibo.it (G.G.); andrea.munari@unibo.it (A.M.); 2 Department of Chemical Science, University of Catania, Viale A. Doria 6, 95125 Catania, Italy; vsiracus@dmfci.unict.it; 3 Organic Synthesis and Photoreactivity Institute, ISOF-CNR, Via Gobetti 101, 40129 Bologna, Italy; massimo.gazzano@cnr.it

**Keywords:** poly(butylene 1,4-cyclohexanedicarboxylate), random copolymers, bio-based polyesters, thermal properties, mechanical properties, barrier properties, structure-property relationship

## Abstract

The present paper describes the synthesis of novel bio-based poly(butylene 1,4-cyclohexane dicarboxylate)-containing random copolymers for sustainable and flexible packaging applications. On one side, the linear butylene moiety has been substituted by glycol subunits with alkyl pendant groups of different length. On the other side, copolymers with different *cis*/*trans* isomer ratio of cyclohexane rings have been synthesized. The prepared samples were subjected to molecular, thermal, diffractometric, and mechanical characterization. The barrier performances to O_2_, CO_2_, and N_2_ gases were also evaluated. The presence of side alkyl groups did not alter the thermal stability, whereas it significantly influences the formation of ordered phases that deeply affect the functional properties, mainly in terms of mechanical response and barrier performance. In particular, the final materials present higher flexibility and significantly improved barrier properties with respect to the homopolymer and most polymers widely employed for flexible packaging. The improvement due to copolymerization was more pronounced in the case of higher co-unit-containing copolymers and for the samples with cyclohexane rings in the *trans* conformation.

## 1. Introduction

Plastic production has grown significantly around the globe. The increased production of plastic requires more fossil resources, which have to be exploited.

The production of plastic reached 335 MT in 2016, with an increase of 3.9% compared to the previous year. In Europe, plastic grows stably, with 39.9% of total 49.9 MT demand in 2016 coming from packaging applications [1]. Barrier films are indispensable for many different applications, ranging from electronic devices to food packaging.

Reducing the amount of finite fossil resources required and mitigating the environmental impact of plastics are becoming very urgent needs, which are leading many industries to look for possible renewable resources.

In addition, efficient management of plastic waste is mandatory in case of plastics employed in packaging, because of the large volumes produced [1].

Bio-based materials are one of the solutions for sustainable packaging, that guarantee product protection and reduced environmental impacts. Consumer concern on environmental impact has also contributed to a demand for more sustainable packaging. Beyond the public perception, sustainable packaging development is driven by retailers and government regulations [2].

In this view, in the last few years, much research had focused on the development of bioplastics showing comparable performances in terms of cost and properties to traditional plastics.

Biodegradable and bio-based plastics are of course the most preferable choice, as they can be derived from natural resources, and are naturally compostable into biomass, carbon dioxide, and water. Within this broad class, poly(butylene *trans*-1,4-cyclohexanedicarboxylate) (PBCE), represents an interesting candidate, due to its smart properties and bio-based origin. As far as the first point is concerned, in particular, it is characterized by high thermal stability and melting temperature, good resistance to heat, light, and humidity [3,4,5,6,7,8], which make it particularly promising for packaging applications. Concerning its bio-based nature, both 1,4-butanediol and *trans*-1,4-cyclohexanedicarboxylic acid may be derived from renewable resources, respectively from succinic acid [9] and bio-based therepthalic acid, in turn obtained from limonene [10].

Recently, our research groups investigated in detail the barrier properties of trans-1,4-cyclohexanedicarboxylate-based polyesters to different gases and in some cases at different temperatures and humidity, which was revealed to be very interesting [4,5,6,7,8].

At the end of last year, we also published a paper describing the effect of side alkyl groups on the final properties of poly(butylene succinate) [11]. In particular, long side alkyl groups were revealed to be an efficient tool to improve the mechanical properties of PBS, reducing its brittleness and rigidity.

On this ground, the aim of the present work is to evaluate the effect on the final properties of PBCE of: *(i)* side alkyl group content, *(ii)* side alkyl group length, and *(iii)* isomerism *cis*/*trans* of cyclohexane aliphatic rings through the study of a novel series of random poly(butylene/2-butyl,2-ethyl-propylene trans-1,4-cyclohexanedicarboxylate) copolymers with different compositions. To shed light on point *(ii)*, the copolymer poly(butylene/neopentyl glycol trans-1,4-cyclohexanedicarboxylate) has been also synthesized. Lastly, a random copolymer containing both the *cis* and *trans* isomers of the cyclohexane ring has been taken into consideration.

To the best of our knowledge, these new copolyesters have been never synthesized before. They have been characterized from the molecular, thermal, mechanical, and barrier properties point of view, in order to envisage their application as sustainable packaging.

## 2. Materials and Methods

### 2.1. Materials

*trans*-1,4-cyclohexanedicarboxylic acid (99%, *cis* 1%) (t-CHDCA), *cis/trans*-1,4-cyclohexanedicarboxylic acid (99%, *cis* 30%) (ct-CHDCA), 1,4-butanediol (BD), neopenthyl glycol (NG), 2-butyl-2-ethyl propanediol (BEPD), and titanium tetrabutoxide (Ti(OBu)_4_) were reagent grade products (Sigma-Aldrich, Saint Louis, MO, USA).

### 2.2. Polymer Synthesis

Poly(butylene *trans*-1,4-cyclohexanedicarboxylate) (PBCE) was synthesized from t-CHDCA and BD, while poly(butylene/2-butyl-2-ethyl-propylene *trans*-1,4-cyclohexanedicarboxylate) (P(BCE_m_BEPCE_n_)) and poly(butylene/neopentyl *trans*-1,4-cyclohexanedicarboxylate) (P(BCE_80_NCE_20_)) random copolymers were prepared starting from different BD/BEPD and BD/NG ratios as glycol moiety, and t-CHDCA as acid monomer (Table 1). For the sake of comparison, the copolymer containing *cis/trans*-1,4-cyclohexanedicarboxylic acid, poly(butylene/2-butyl-2-ethyl-propylene *cis/trans*-1,4-cyclohexanedicarboxylate) (P(BCE_65_BEPCE_35_-ct)), was also synthesized. In all cases, 100% glycol molar excess was used with respect to diacid content (Table 1).

All the reactions were carried out in bulk, employing titanium tetrabutoxide as catalyst (about 150 ppm of Ti/g of polymer) in a 200 mL glass reactor, with a thermostatted silicon oil bath; temperature and torque were continuously recorded during polymerization. The polymers were obtained according to the usual two-stage polymerization procedure. In the first stage, under pure nitrogen flow, the temperature was set at 180 °C and kept constant until more than 90% of the theoretical amount of water was distilled off (about 120 min). In the second stage, the pressure was progressively reduced to 0.1 mbar, in order to facilitate the removal of the glycol excess, and the temperature was risen to 220 °C. The syntheses were stopped after about four additional hours (up to a constant value of the measured torque).

**Figure 1:**
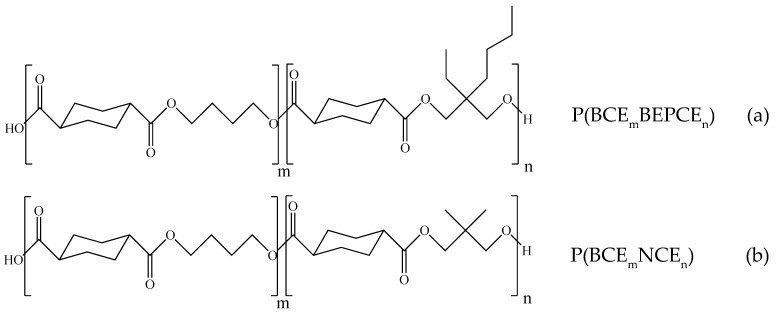
Chemical structures of P(BCE_m_BEPCE_n_) (**a**) and P(BCE_m_NCE_n_) (**b**) random copolymers.

Light colored materials with very high yield (Table 1) were obtained by using the described procedure. The molecular formula of the PBCE-based random copolymers is reported in Figure 1.

### 2.3. Film Preparation

Films were obtained by compression moulding the polymer pieces between two Teflon plates with an appropriate spacer at a temperature *T* = *T*_m_ + 40 °C for 2 min under a pressure of 2 ton/m^2^ (Carver C12, laboratory press, Wabash, Indiana USA). Afterwards, the films were cooled (≈65 °C·min^−1^) to room temperature, keeping the applied pressure.

Prior to the characterization, the films were stored under vacuum at room temperature for at least three weeks to reach thermal equilibrium.

The film thickness was determined using a Digital Dial Indicator (MarCator 1086 type, Mahr GmbH, Esslingen, Germany) connected to a PC using the Sample Thickness Tester DM-G software (Mahr GmbH, Esslingen, Germany). The reading was made measuring a minimum, a maximum, and an average value. The results represent the mean value thickness of three experimental tests run at 10 different points on the polymer film surface at room temperature.

### 2.4. Molecular and Thermal Characterization

Molecular characterization was performed by means of proton and carbon nuclear magnetic resonance spectroscopy (^1^H-NMR and ^13^C-NMR) at room temperature, employing a Varian Inova 400-MHz instrument (Agilent, Technologies, Palo Alto, CA, USA). Operating settings: (i) ^1^H-NMR: relaxation delay of 0 s, acquisition time of 1 s, and up to 100 repetitions and relaxation delay of 1 s; (ii) ^13^C-NMR: acquisition time of 1 s, up to 700 repetitions and a full decoupling mode. The samples were dissolved in chloroform-d with 0.03 v% tetramethylsilane.

Molecular weights were evaluated by gel-permeation chromatography (GPC) at 30 °C using a 1100 HPLC system (Agilent Technologies, Santa Clara, CA, USA) equipped with PLgel 5-μm MiniMIX-C column (Agilent Technologies, Santa Clara, CA, USA). A refractive index was employed as detector. Chloroform was used as eluent with a 0.3 mL/min flow and sample concentrations of about 2 mg/mL. A molecular weight calibration curve was obtained with polystyrene standards in the range of 2000–100,000 g/mol.

Thermogravimetric analysis (TGA) was carried out under nitrogen atmosphere using a Perkin Elmer TGA7 apparatus (gas flow: 40 mL/min, Waltham, MA, USA) at 10 °C/min heating rate up to 900 °C.

Calorimetric measurements were conducted by using a Perkin Elmer DSC7 instrument (Waltham, MA, USA). In the typical setup, the external block temperature control was set at −70 °C and weighed samples of c.a. 10 mg were heated up to 190 °C at a rate of 20 °C/min (first scan), held there for 5 min, and then rapidly quenched by immersing the pans in liquid nitrogen. Finally, they were reheated from −70 °C to a temperature well above the melting point of the sample at a heating rate of 20 °C/min (second scan). The glass-transition temperature (*T*_g_) was taken as the midpoint of the heat capacity increment Δ*c*_p_ associated with the glass-to-rubber transition. The cold crystallization temperature (*T*_cc_) and the disordering temperature (*T*_I_ and *T*_II_) were determined as the peak value of the exothermal and endothermal phenomena in the DSC curve, respectively. The specific heat increment Δ*c*_p_, associated with the glass transition of the amorphous phase, was calculated from the vertical distance between the two extrapolated baselines at the glass transition temperature. The heat of cold crystallization (Δ*H*_cc_) and the heat of disordering (Δ*H*_I_ and Δ*H*_II_) of the ordered phase were calculated from the total areas of the DSC exotherm and endotherm, respectively.

### 2.5. Wide-Angle X-ray Analysis

X-ray diffraction patterns were obtained with Cu*K*α radiation in reflection mode by means of an X’Pert PANalytical diffractometer (PANalytical, Almelo, The Netherlands) equipped with a fast X’Celerator detector, 0.1° step, 100s/step. The samples were analysed in the form of films. The indices of crystallinity (*X*_c_) were calculated from the X-ray diffraction profiles by the ratio between the crystalline diffraction area (*A*_c_) and the total area of the diffraction profile (*A*_t_), *X*_c_ = *A*_c_/*A*_t_. The crystalline diffraction area was obtained from the total area of the diffraction profile by subtracting the amorphous halo. The incoherent scattering was taken into consideration. The unit cell parameters were calculated by whole pattern fitting using Powder Cell 2.3 for Windows [12].

### 2.6. Stress-Strain Measurements

The tensile testing of PBS and its random copolymers was performed using a Zwick Roell Texture machine (Ulm, Germany), equipped with rubber grip and controlled by computer. A pre-load of 1 MPa with a 5 mm/min speed, on a 500 N load cell, was used. 5 × 50 mm^2^ films with an initial grip separation of 23 mm were employed. The stress-strain measurements were performed with a crosshead speed of 50 mm/min. Five different samples from the same film were tested for each copolymer composition and the results were provided as the average value ± standard deviation. All tests were carried out in accordance with ASTM D638 procedure.

### 2.7. Gas Transport Measurements

The determination of the gas barrier behavior was performed by a manometric method using a Permeance Testing Device, type GDP-C (Brugger Feinmechanik GmbH, München, Germany), according to ASTM 1434-82 (Standard test Method for Determining Gas Permeability Characteristics of Plastic Film and Sheeting), DIN 53 536 in compliance with ISO/DIS 15 105-1 and according to Gas Permeability Testing Manual (Registergericht München HRB 77020, Brugger Feinmechanik GmbH).

After a preliminary high vacuum desorption of the upper and lower analysis chambers, the upper chamber was filled with the gas test at ambient pressure. A pressure transducer set in the lower chamber continuously records the increasing gas pressure as a function of time [13,14]. The gas transmission rate (GTR) was determined considering the increase in pressure in relation to the time and the volume of the device. All the measurements have been carried out at room temperature of 23 °C. The operative conditions were: gas stream of 100 cm^3^·min^−1^; 0% RH of gas test, food grade; sample area of 78.5 cm^2^ (standard measurement area). Gas transmission measurements were performed at least in triplicate and the mean value is presented.

## 3. Results and Discussion

### 3.1. Molecular Characterization

The molecular characterization data of the polymers under investigation are reported in Table 2: all the samples were characterized by high and similar molecular weights, proving that no significant thermal degradation reactions occurred during polymerization.

**Figure 2:**
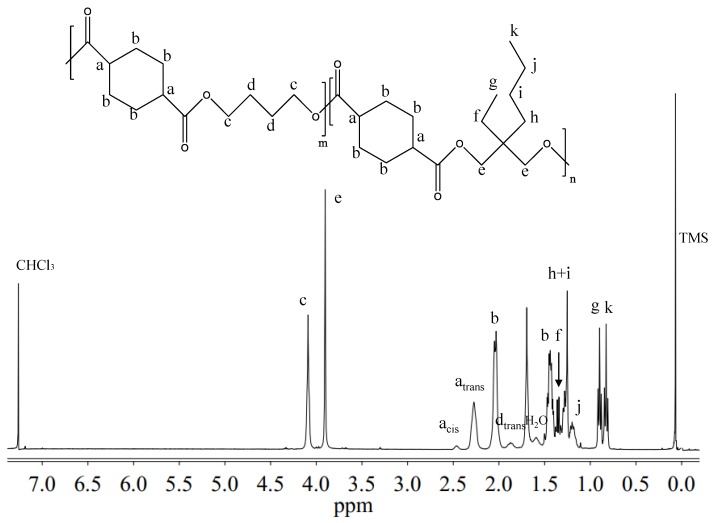
^1^H-NMR spectrum of P(BCE_65_BEPCE_35_) with resonance assignments.

^1^H-NMR and ^13^C-NMR analysis have been carried out to *(i)* verify the chemical structure, *(ii)* calculate the actual composition, and *(iii)* calculate the degree of randomness (b). As an example, Figure 2 reports the ^1^H-NMR spectrum of P(BCE_65_BEPCE_35_) with the corresponding resonance assignments.

**Figure 3:**
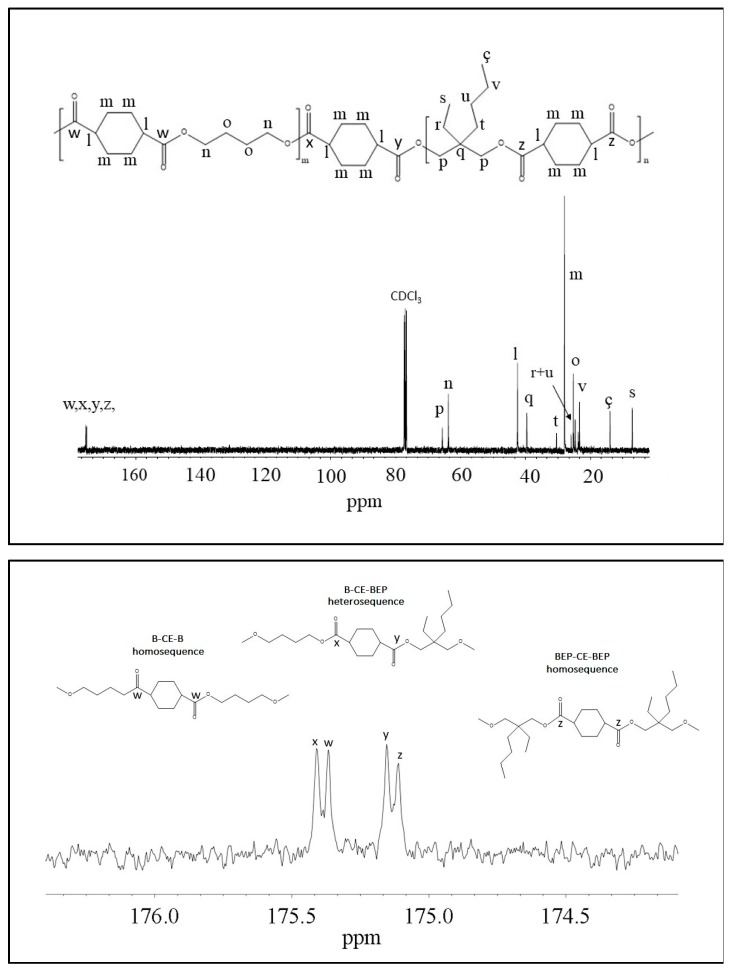
(**Top**): ^13^C-NMR spectrum of P(BCE_65_BEPCE_35_) with resonance assignments; (**Bottom**): Enlargement of ^13^C-NMR spectrum in the region 176.50–174 ppm, together with the schematic representation of B-CE-B, B-CE-BEP, and BEP-CE-BEP triads.

The chemical structure of the copolymer is confirmed, since no additional peaks were found in the spectrum. The copolymer composition was determined from the relative areas of the resonance peak of the *c* protons of the butylene sub-unit, located at 4.11 ppm and of the signal at 3.90 ppm corresponding to the *e* protons of the butyl-ethyl propylene moiety for P(BCE_m_BEPCE_n_) (see Figure 2). In the case of P(BCE_80_NCE_20_), the signal of neopentyl subunit at 3.85 ppm has been used for calculating the composition (data not shown). For all the copolymers, the actual composition is close to the feed one (see Table 2), proving a good control in the polymerization process. The degree of randomness (b) has been calculated by ^13^C-NMR spectroscopy. In Figure 3, as example, is reported the ^13^C-NMR spectrum of P(BCE_65_BEPCE_35_) with the peaks assignments (top) and the magnification of the region in between 176.50 and 174 ppm, where the signals due to the ester groups carbons are located (bottom). In this region, together with the signals of the ester carbons at 175.38 and 175.13 ppm, corresponding to the B-CE-B (*w* carbon) and BEP-CE-BEP (*z* carbon) triads, respectively, two additional peaks can be detected. These signals refer to the B-CE-BEP and BEP-CE-B (*x* and *y* carbons) triads, due to transesterification reactions. The degree of randomness b has been calculated from the intensity of the *w*, *z*, *x*, and *y* peaks.

It is worth noting that *b* is equal to 1 for random copolymers, equal to 2 for alternate copolymers, equal to 0 for a mixture of two corresponding homopolymers and 0 < *b* < 1 for block copolymers. The degree of randomness was calculated according to Equation (1):

(1) $$b=P_{B-\mathrm{BEP}}+P_{\mathrm{BEP}-B}$$

where B-BEP and BEP-B are the probability of finding a B unit next to a BEP one and the probability of finding a BEP unit close to a B one, respectively.

In turn, the two probabilities can be expressed as Equation (2):

(2) $$P_{B-\mathrm{BEP}}=\frac{I_{x}}{I_{x}+I_{w}};\;P_{\mathrm{BEP}-B}=\frac{I_{y}}{I_{y}+I_{z}}$$

where, *I*_w_, *I*_x_, *I*_y_, *I*_z_ represent the integrated intensities of the resonance peaks of the B-CE-B, B-CE-BEP, BEP-CE-B, and BEP-CE-BEP triads, respectively (Figure 3 bottom).

For all the copolymers, the calculated b is practically equal to 1. Therefore, we can conclude that the experimental conditions adopted allowed us to synthesized copolymers with a random distribution of sequences.

### 3.2. Thermal Characterization

**Figure 4:**
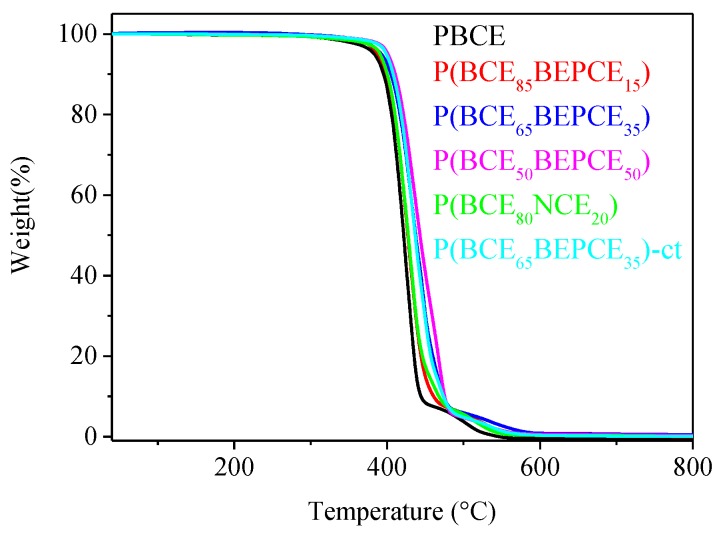
TGA traces of PBCE homopolymer and P(BCE_m_BEPCE_n_) and P(BCE_80_NCE_20_) random copolymers.

The thermal stability of the samples under investigation has been analysed by TGA under nitrogen flow. The temperatures of 5% weight loss (*T*_5%_) and maximum weight loss rate (*T*_max_) have been collected in Table 3. In all cases, the weight loss takes place in one step and is 100% (see Figure 4). All the copolymers under study show very good thermal stability, with the weight loss starting above 380 °C. Both *T*_5%_ and *T*_max_ increase in the copolymers evidencing the thermal degradation takes place at higher temperature. This result is probably due to the presence of ramifications on the carbon in the β position with respect to the ester oxygen that hinders the β-scission process. The observed trend appears to be correlated to copolymer composition.

**Figure 5:**
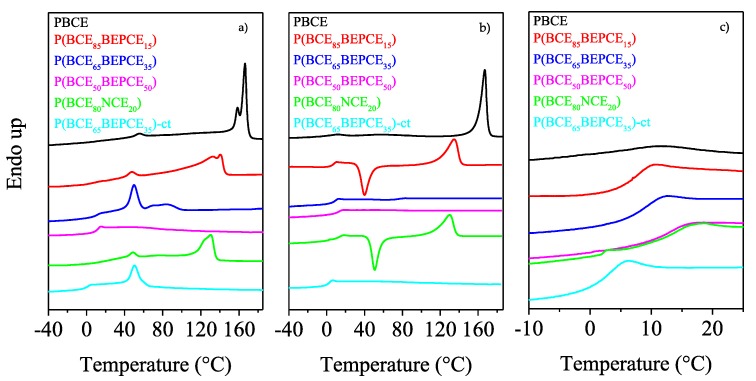
Calorimetric curves (heating rate: 20 °C/min) of PBCE, P(BCE_m_BEPCE_n_), and P(BCE_80_NCE_20_) copolymers. (**a**) 1st scan and (**b**) 2nd scan after quenching from the melt; (**c**) magnification in the *T*_g_ region of 2nd scan.

The calorimetric curves and the relative thermal data of the samples under investigation are reported in Figure 5 and Table 3, respectively. Regarding the calorimetric study, we can exclude an influence of molecular weight on the glass transition and melting of the synthesized polymers due to the samples being characterized by high and similar *M*_n_.

As one can see from the first calorimetric scan (Figure 5a), all the samples, except for P(BCE_50_BEPCE_50_), are semicrystalline, with the corresponding DSC traces characterized by the presence of a baseline deviation associated to the glass to rubber transition, followed by endothermic peaks at higher temperatures related to the melting of ordered portions. The P(BCE_50_BEPCE_50_) sample just shows the glass-to-rubber transition at 11 °C.

Concerning the semicrystalline samples, two different endothermic phenomena can be seen: the first one at intermediate and fixed temperature, around 50 °C, whose intensity increases with the comonomeric unit content, from now on referred to as peak I; the second one at higher temperature, characterized by a double peak, whose position and area progressively decrease with the co-units amount, from now on referred to as peak II.

The evolution of peak II in the copolymers under study is typical of most copolyester systems, for which, as the co-units amount increases, the formation of a crystalline phase with lower degree of perfection occurs. As concerns the multiple nature of peak II, it can be ascribed to melting/crystallization processes of low ordered crystals typical of copolymer system in which the co-units hinder the crystallization process, or can be due to the presence of different crystal lattices [15,16,17,18].

On the other side, Peak I shows a peculiar behavior not typical of copolyesters, since it is located at the same temperature regardless of the composition and increases in intensity when the co-unit content rises. The calorimetric curve of the copolymer containing *cis/trans*-1,4-cyclohexanedicarboxylate moieties, P(BCE_65_BEPCE_35_)-ct, is very similar to that of P(BCE_65_BEPCE_35_) in which just *trans*-1,4-cyclohexanedicarboxylate subunits are present, the only difference being represented by the low melting peak at 85 °C in P(BCE_65_BEPCE_35_) trace. The absence of this endotherm in P(BCE_65_BEPCE_35_)-ct can be explained by the additional crystallization hindering of the *cis/trans* isomerism in the acid subunit that further reduces the structural regularity in the macromolecular chain.

X-ray diffraction analysis can provide more elements to identify the origin of these endothermic phenomena: peak I and peak II.

As it is well known, a partially crystalline material usually exhibits different glass transition behaviour than completely amorphous analogous. In fact, although some conflicting results are reported in the literature [19], crystallinity usually acts like crosslinking and raises *T*_g_ values through its restrictive effect on the segmental motion of amorphous polymer chains. Therefore, to study the influence of chemical structure on the glass transition of random copolymers, the phenomenon should be examined in the total absence of crystallinity. In this view, all the samples under investigation were subjected to rapid cooling (quenching) from the melt (see the Experimental section for the details). The DSC curves and the thermal characterization data of the so-treated samples are reported in Figure 5b and collected in Table 3 as a function of co-unit content.

The effect of copolymerization on the thermal behaviour of the PBCE homopolymer is evidenced by the calorimetric curves obtained after melt quenching. As one can see, the PBCE curves of I and II scans are practically the same (see Figure 5a,b), evidencing that, due to its high crystallization rate, this homopolymer cannot be quenched in the amorphous state under the experimental conditions adopted. On the contrary, the DSC traces of all the copolymers change after melt quenching in liquid nitrogen. Firstly, the glass transition phenomenon becomes more evident, as a consequence of an increased amorphous phase amount in the quenched samples. Secondly, a different crystallization capability can be evidenced as the glycol co-unit content is increased. In particular, for the samples containing up to 20 mol% of glycol co-units, P(BCE_85_BEPCE_15_ e P(BCE_80_NCE_20_), the corresponding DSC traces are characterized by the glass to rubber transition step, an exothermic peak and an endothermic one, located at higher temperatures. This kind of DSC trace is typical of materials that, once the glass transition temperature is exceeded, are able to crystallize in the temperature window in between *T*_g_ and *T*_m_ and then undergo melting of the crystals developed during the heating. Moreover, with the areas under the two peaks equal (Δ*H*_cc_ = Δ*H*_II_), we can assert that the copolymers had been quenched into the amorphous phase, with the presence of BEPCE and NCE co-units along the PBCE chains hindering its crystallization capability. This effect is even more evident in the copolymers with higher glycol co-unit content, P(BCE_65_BEPCE_35_) e P(BCE_50_BEPCE_50_), for which just the glass to rubber transition step has been detected in the second scan DSC trace. P(BCE_65_BEPCE_35_)-ct has the same phase behavior of P(BCE_65_BEPCE_35_) e P(BCE_50_BEPCE_50_), i.e., is completely amorphous after melt quenching (the corresponding DSC trace evidences only the glass transition phenomenon).

The co-unit nature has a direct effect also on the polymer chain mobility as indicated by the *T*_g_ and the corresponding Δ*c*_p_ values reported in Table 3, and as shown by the DSC curves magnification of Figure 5c. As already reported in the literature, the presence of alkyl pendant groups hinders the rotation around the C–C σ bond, beacause of their high steric hindrance, reducing the macromolecular mobility (*T*_g_ rises). However, if the pendant groups are long enough, they are able to exert an internal plasticizing effect, which leads to a decrease of the *T*_g_ value. The entity of this latter effect is proportional to the length of the side alkyl group. In the copolymers under study, both these opposite effects are supposed to influence chain mobility, i.e., *T*_g_ position.

To analyze the effect of the presence of the different co-units, both in the glycol and acid mioeties, on the *T*_g_ of PBCE homopolymer, in Figure 5c, we have reported the temperature region at which the glass to rubber transition occurs.

As one can see, the *T*_g_ step, rather low for the semicrystalline PBCE, increases in height in the copolymers, as a consequence of the presence of the only amorphous phase after melt quenching. Therefore, any effect of crystal phase on glass transition temperature can be excluded: the *T*_g_ position will be solely due to chain mobility. It is interesting to note that for all samples, except for P(BCE_65_BEPCE_35_)-ct, despite the absence of crystalline domains, the glass to rubber transition step progressively moves towards higher temperature (see also Table 3), similar to other copolymeric systems previously investigated [20,21]: the chain stiffening, due to the steric hindrance of the alkyl pendants introduced by copolymerization, prevails over their plasticizing effect.

For the NCE co-unit containing copolymer, P(BCE_80_NCE_20_), the balance of the steric hindrance and plasticizing effect of side pendants is different, and determines the same *T*_g_ increase observed for P(BCE_m_BEPCE_n_) with lower co-unit weight fraction. In fact, as one can see in Figure 5c, the calorimetric trace of P(BCE_80_NCE_20_) practically overlaps that of P(BCE_50_BEPCE_50_) (see Table 3). This result indicates that in the case of NCE comonomeric units, the internal plasticizing effect is minor, probably due to the lower length of the neopentyl side chains.

The only case in which a decrease in *T*_g_ value with respect to PBCE homopolymer has been observed is for the copolymer with *cis/trans*-1,4-cyclohexanedicarboxylate moieties, P(BCE_65_BEPCE_35_)-ct. The different ring isomerism causes a decrease in the chain symmetry and probably limits interchain interactions with a consequent lowering of the glass transition temperature.

### 3.3. Structural Characterization

**Figure 6:**
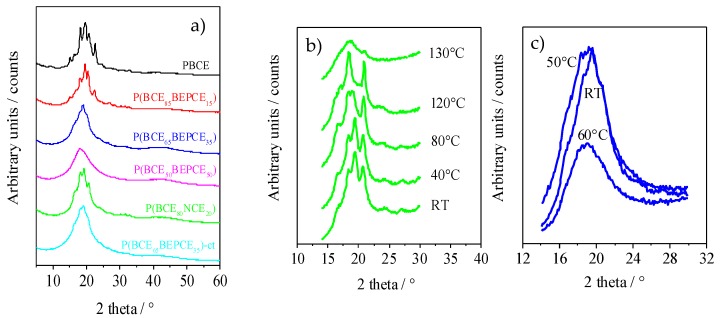
(**a**) XRD profiles at room temperature of all the samples under study; (**b**,**c**) XRD profiles at different temperatures for the compression molded films of P(BCE_80_NCE_20_) and P(BCE_65_BEPCE_35_), respectively.

The XRD patterns of the polymers of this study are reported in Figure 6a. The results, corresponding to the films of the calorimetric I scan traces, show that copolymerization induces wide crystallinity variation. The PBCE homopolymer is characterized by a more defined profile containing several peaks located at 9.3°, 15.1°, 16.6°, 18.2°, 19.3°, 20.7°, and 22.7°. As one can see from Figure 6a, copolymerization causes a decrease in the intensity of XRD reflections even for low percentages of co-unit content. This effect is more pronounced for some peaks, particularly the ones at 9.3°, 15.1°, and 22.7°, that, still present in P(BCE_85_BEPCE_15_), disappear in the P(BCE_80_NCE_20_) copolymer containing a higher co-unit content, for which just the peaks at 16.6°, 18.2°, 19.3°, and 20.7° can be detected. These reflections, reduced consistently in intensity, are also the only ones visible in the copolymers with 35 mol% of BEPCE segments: P(BCE_65_BEPCE_35_) and P(BCE_65_BEPCE_35_)-ct. The disappearance of some peaks by copolymerization is not surprising, taking into account that previous studies have highlighted the polymorphic nature of PBCE homopolymer [6,22]. In this view, the selective reduction of the peaks 9.3°, 15.1°, and 22.7°, can be associated with the disappearance of an ordered phase whose formation is progressively compromised by the insertion of BEPCE and NCE segments in the main polymer chain. In the case of the P(BCE_50_BEPCE_50_) copolymer, no clear diffractometric reflections can be detected in the relative WAXS spectrum.

Moreover, the XRD results show no evidence of co-crystallization, either in P(BCE_80_NCE_20_) or in P(BCE_m_BEPCE_n_). This result is in agreement with the calorimetric data showing a lowering of *T*_I_ proportional to the quantity of co-unit and independent on its nature.

Finally, the comparison of P(BCE_65_BEPCE_35_) and P(BCE_65_BEPCE_35_)-ct shows that the different isomerism of cyclohexane ring does not affect the nature of the crystalline phase developed.

The XRD measurements carried out at room temperature do not clarify the complex scenario evidenced for this copolyester system by DSC analysis. Consequently, temperature scanning diffractometric experiments have also been performed to follow the structural evolution of the polymers under study. In Figure 6b, the XRD spectrum of P(BCE_80_NCE_20_) at different temperatures is reported. As one can see, as temperature increases, reflections at 18.2° and 20.7° arise and progressively grow at the expense of the peaks at 16.6° and 19.3°. The disappearance of these occurs at a temperature close to that of the endothermic peak I evidenced by calorimetric analysis (Figure 5a), that is practically the only one present in the DSC traces of P(BCE_65_BEPCE_35_) and of P(BCE_65_BEPCE_35_)-ct copolymers. By further increasing the temperature, an improvement of peak resolution can be observed, suggesting that the double DSC peak II could be due to melting/crystallization of low ordered crystals.

In Figure 6c, WAXS patterns of P(BCE_65_BEPCE_35_) at different temperatures are reported. It is worth noting that, at room temperature, this sample does not show defined peaks nor the typical bell shape of diffractometric amorphous halo. On the contrary, its corresponding pattern is pretty sharp and characterized by the presence of a broad peak that becomes less defined at 50 °C and suddenly disappears when the temperature reaches 60 °C. Simultaneously, at 60 °C, the XRD pattern presents the typical amorphous halo.

Comparing the evolution of the diffractometric profile measured at 50 °C, which is certainly not characteristic of a semicrystalline material, to the pattern at 60 °C, which is on contrary the typical pattern of a completely amorphous polymer, the presence of a 2D-ordered phase in the polymers under study can be hypothesized. As a matter of fact, in the wide group of main chain mesogenic-containing polymer liquid crystals (PLCs), there are different examples including 1,4-cyclohexane moieties as mesogenic groups [23].

### 3.4. Mechanical Characterization

**Figure 7:**
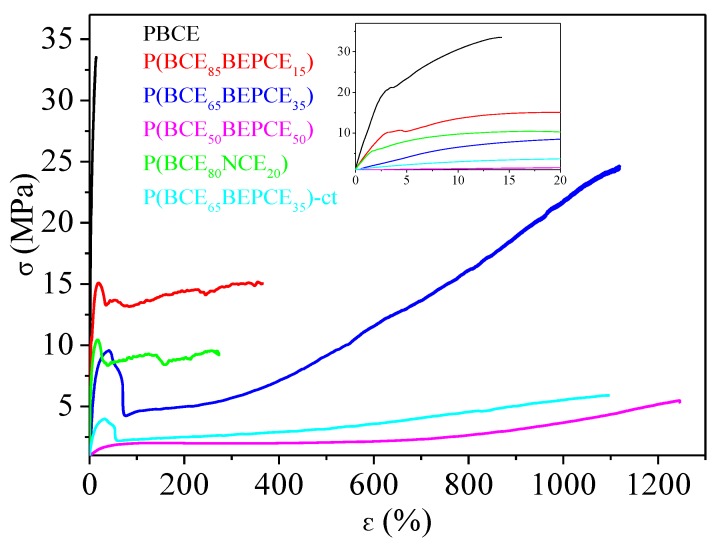
Stress-strain curves of PBCE, P(BCE_m_BEPCE_n_), and P(BCE_80_NCE_20_) random copolymers; In the inset: magnification of the low σ vs. ε region.

To test the mechanical performances of the materials synthesized, tensile measurements have been performed. The stress strain curves are shown in Figure 7 and the mechanical data (elastic modulus *E*, stress at break σ_b_ and elongation at break ε_b_) are listed in Table 4.

As one can see, the mechanical characterization has been performed on all samples, even on P(BCE_50_BEPCE_50_). This fact is quite surprising taking into account that this copolymer at room temperature is in its rubbery state and both calorimetric and diffractometric analyses do not evidence the presence of crystalline structures. Considering this situation, the possibility of obtaining a freestanding film of P(BCE_50_BEPCE_50_) could be attributed to the presence of a 2D-ordered phase, usually referred to as mesophase, developing in the material.

Previous studies have demonstrated that in main chain mesogenic-containing polymer liquid crystals (PLCs) with an even number of flexible –CH_2_– groups, a smectic liquid crystal mesophase develops [24,25]. The corresponding isotropization is revealed in the DSC curve as a low intensity endothermic peak at a temperature between *T*_g_ and *T*_m_. This should be the case for the homopolymer PBCE and its copolymers containing a higher amount of even number methylene group subunits (butylene moiety) with respect to the odd number –CH_2_– glycol unit, i.e., BEP moieties (P(BCE_85_BEPCE_15_ and P(BCE_65_BEPCE_35_). These polymers, in fact, show an endothermic peak at about 50 °C that, as mentioned before, is not directly attributable to a classic 3D-ordered structure, being independent of composition and appearing at the same temperature regardless of the co-unit content.

On the other hand, for PLCs containing an odd number of –CH_2_– groups, the formation of a nematic mesophase is favoured [24,25]. This kind of 1D-ordered phase can be also detected by DSC analysis, but the relative peak is located above T_m_ and the corresponding isotropization heat is lower than that of smectic mesophase. This could be the case for P(BCE_50_BEPCE_50_), which contains equimolar amounts of glycol sub-unit with even number of methylene groups (butylene subunits) and an odd number of –CH_2_– (BEP) moieties.

The PBCE homopolymer appeared to be the most rigid material among those investigated, being characterized by the highest elastic modulus and stress at break, accompanied by the lowest elongation at break. The introduction of BEPCE co-units affects the mechanical response of PBCE homopolymer, causing the decrease of both E and σ_b_. The entity of variation is a function of copolymer composition: the higher the co-unit amount, the larger the effect. This result is probably related to the gradual reduction of the crystallinity degree, X_c_, in the copolymers with respect to the homopolymer. In particular, for the highest BEPCE co-units content P(BCE_50_BEPCE_50_), E decreases by more than two orders of magnitude, while σ_b_ is ten times lower. The presence of BEPCE comonomeric units also leads to an increase of ε_b_ from 19% for PBCE up to 1290% for P(BCE_50_BEPCE_50_).

By comparing P(BCE_85_BEPCE_15_) and P(BCE_80_NCE_20_) copolymers, it is observed that these samples have very similar composition but different glycol co-units and it is possible to evaluate the effect of side chain length on the mechanical response of the final material. In particular, as one can see, P(BCE_80_NCE_20_), although less crystalline, has an elastic modulus comparable to that of P(BCE_85_BEPCE_15_) and lower elongation at break. This result can be attributed to the minor plasticizing effect of the shorter side chains of the NCE co-units.

On the other side, comparison of P(BCE_65_BEPCE_35_) and P(BCE_65_NCE_35_)-ct copolymers, which have very similar compositions but different acid co-units, allows us to determine the effect of *cis/trans* isomerism of the aliphatic cyclohexane. As already highlighted by the calorimetric and diffractometric results, the two copolymer films have the same degree of crystallinity, *X*_c_, and this explains the same elongation at break ε_b_ of the two materials. However, the sample containing *cis/trans*-1,4-cyclohexanedicarboxylate subunits shows lower elastic modulus and tensile stress. This evidence is probably related to the reduced structural symmetry that lowers the interactions between the different polymer chains and, consequently, facilitates their sliding under stretch.

### 3.5. Gas Barrier Performance

Gas barrier performances to dry N_2_, O_2_, and CO_2_ gases were evaluated at 23 °C, with the aim of enhancing the lifetime of the devices.

**Figure 8:**
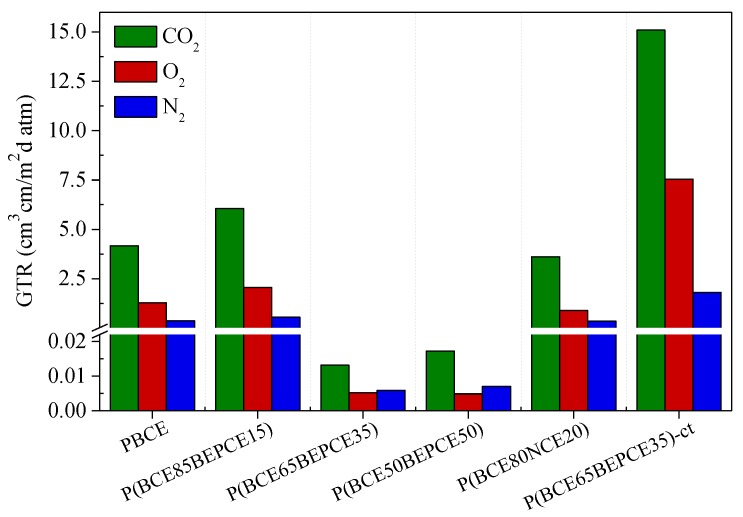
GTR of N_2_, O_2_, and CO_2_ through PBCE, P(BCE_m_BEPCE_n_), P(BCE_80_NCE_20_), and P(BCE_65_BEPCE_35_)-ct films at 23 °C.

The permeability values, expressed as Gas Transmission Rate (GTR), are collected in Table 4 and shown in Figure 8 for the films under study.

The results show that the introduction of low amount of BEP glycol co-units in the main PBCE polymer chain causes a slight increase of gas permeability. On the contrary, by increasing the percentage of comonomer (35 and 50 mol%), an outstanding improvement of the barrier effect is achieved: the measured GTR value is 50 times lower for N_2_ and 250 times decreased for O_2_ and CO_2_. The result is even more surprising if we consider that the P(BCE_65_BEPCE_35_) copolymer is much less crystalline than the PBCE homopolymer and the P(BCE_50_BEPCE_50_) sample is even amorphous. Consequently, the very low GTR values cannot be explained, on the basis of the higher crystallinity degree of these materials. Moreover, considering that in the operative conditions, both copolymers P(BCE_65_BEPCE_35_) and P(BCE_50_BEPCE_50_) are in the rubbery state (*T*_g_ < 23 °C), the extraordinary barrier performances cannot be attributable to the glassy state that, as well known, shows a free volume lower than the corresponding rubbery phase. In this view, the experimental data could probably be explained as being due to the polymer chains’ ability to develop a 2D-orderd structure, generally defined as mesophase, which hinders the gas passage. This kind of arrangement is due to the simultaneous presence in the polymer chain of rigid units (aliphatic cyclohexane ring) alternating with flexible segments (aliphatic glycolic moiety). Therefore, the presence of this 2D-organized phase, evidenced by calorimetric and diffractometric experiments and also suggested by the mechanical response obtained, could be responsible for the outstanding barrier properties of these materials, analogous to what happens in crystalline liquids.

By comparing the two copolymers with similar composition but different side chains, P(BCE_85_BEPCE_15_) and P(BCE_80_NCE_20_), the effect of chain length can be evidenced. The short branch P(BCE_80_NCE_20_) copolymer shows better barrier performance. This result is in line with the higher macromolecular rigidity revealed by calorimetric analysis for the NCE containing copolyester. Again, the result observed is not explainable on the basis of crystallinity degree, with the P(BCE_80_NCE_20_) sample being less crystalline than P(BCE_85_BEPCE_15_). A possible reason for the experimental data could be the less effective arrangement that can be achieved in the copolymer containing longer and more sterically encumbering ramifications. On the contrary, the shorter ramifications in the P(BCE_80_NCE_20_) sample favor a more efficient macromolecular chain packing that can better hamper the gas molecules’ transit.

It is also interesting to note how the isomerism of the cyclohexane ring has a remarkable effect on gas barrier properties. In particular, the presence of a *cis* isomer together with the *trans* one in P(BCE_65_BEPCE_35_)-ct determines a substantial increase of GRT values. To explain this effect, one could consider that, unlike the *trans* isomer rings that tend to assume the chair conformation in the polymer chain, the rings with *cis* isomerism are more stable in the boat conformation. Accordingly, in the copolymer with an equimolar mixture of *cis* and *trans* acid sub-units, the stacking of the mesogenic aliphatic cyclohexane rings, and thus the formation of 2D ordered domains, will be limited between rings of the same type. This situation leads to less efficient packing and therefore to higher free volume, as also highlighted by the calorimetric analysis (lower *T*_g_), with a consequent increase of gas molecule permeability for P(BCE_65_BEPCE_35_)-ct.

Finally, for all samples under consideration, CO_2_ is the gas that spreads faster through the films despite its larger dimension. This evidence can be related to the lower CO_2_ solubility in the highly hydrophobic polymer matrix.

**Figure 9:**
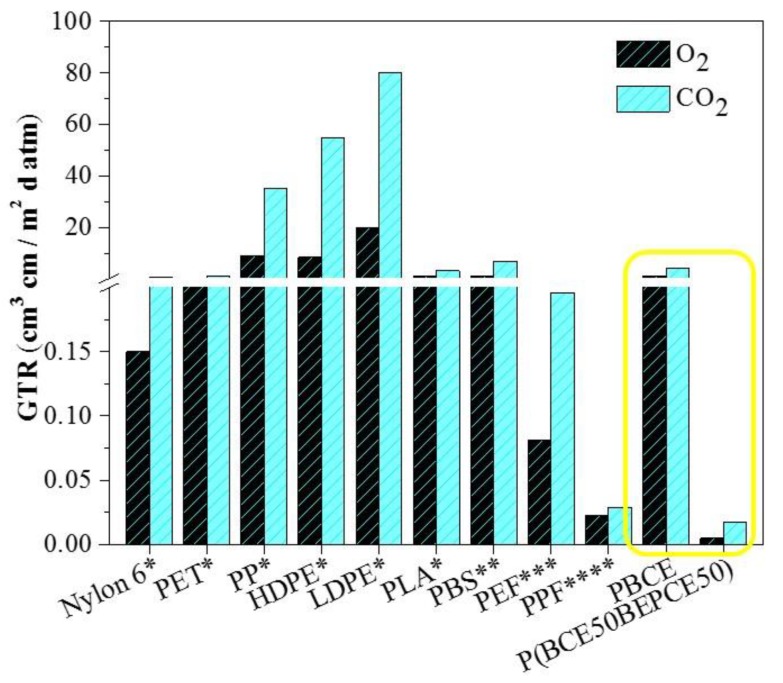
Gas transmission rate of O_2_ and CO_2_ gases for PBCE and P(BCE_50_BEPCE_50_) and for some traditional petrochemical-based polymeric packaging materials and for some biopolymers (poly(ethylene terephthalate) PET, polypropylene PP, high density polyethylene HDPE, low density polyethylene LDPE, polylactic acid PLA, poly(butylene succinate) PBS, poly(ethylene furanoate) PEF, poly(propylene furanoate) PPF) from References [26,27,28,29,30].

Last but not least, the PBCE homopolymer and the P(BCE_50_BEPCE_50_) random copolymer, the latter having the best performance among the polymers investigated in the present paper, have been compared with some common petrochemical-based polymeric packaging materials and some biopolymers (see Figure 9).

As it can be seen, P(BCE_50_BEPCE_50_) exhibits excellent barrier properties, being by far the highest performing material among those considered. Whilst this comparison is far from being exhaustive, it can be considered meaningful to highlight the potential of these new materials for use as high barrier films.

## 4. Conclusions

New high molecular weight PBCE-based copolyesters containing side aliphatic chains of different lengths were successfully prepared by a simple, solvent-free process, which can be of interest for industry. The new materials appeared to be characterized by interesting properties in view of a possible application in flexible packaging. The final properties can be tuned by playing with copolymer composition, the length of aliphatic side chain, and the *cis/trans* isomerism of the aliphatic ring.

In the context of films for flexible packaging, the two copolymers richest in BEPCE co-units are particularly noteworthy, being characterized by elastomeric behavior and exceptional barrier properties to oxygen and carbon dioxide. The outstanding barrier properties are hypothesized to be related to the presence of an ordered 2D phase, generically defined as a mesophase, particularly important for the copolymer P(BCE_50_BEPCE_50_). This latter material can indeed be processed as a freestanding flexible film despite being amorphous and having a *T*_g_ below room temperature. Its barrier performance is significantly better than those of poly(ethylene 2,5-furanoate) and poly(propylene 2,5-furanoate), two bio-based high performant barrier materials.

In conclusion, the new polymers proposed in the present study can be considered interesting candidates for sustainable packaging. 

## Figures and Tables

**Table 1:** Starting reagent amounts, molar diol excess, and yield data for PBCE, P(BCE_m_BEPCE_n_), and P(BCE_80_NCE_20_) random copolymers.

Polymer	CHDCA mmol	BD mmol	BEPD mmol	NG mmol	Diol Excess mol%	Yield %
**PBCE**	29	58	-	-	100	95
**P(BCE_85_BEPCE_15_)**	29	49	9	-	100	95
**P(BCE_65_BEPCE_35_)**	29	38	20	-	100	96
**P(BCE_50_BEPCE_50_)**	29	29	29	-	100	96
**P(BCE_80_NCE_20_)**	29	46	-	12	100	95
**P(BCE_65_BEPCE_35_)-ct**	29	38	20	-	100	96

**Table 2:** Molecular characterization data of PBCE, P(BCE_m_BEPCE_n_) and P(BCE_80_NCE_20_) random copolymers.

Polymer	BCE mol% feed	BCE mol% ^1^H-NMR	BCE wt % ^1^H-NMR	*cis* mol% ^1^H-NMR	b ^13^C-NMR	M_n_ g/mol gpc	D gpc
**PBCE**	100	100	100	2	-	44,300	2.3
**P(BCE_85_BEPCE_15_)**	85	85	81	2	1.01	45,700	2.3
**P(BCE_65_BEPCE_35_)**	65	65	59	3	1.01	45,900	2.5
**P(BCE_50_BEPCE_50_)**	50	51	45	3	0.98	46,600	2.2
**P(BCE_80_NCE_20_)**	80	80	79	2	0.99	46,300	2.2
**P(BCE_65_BEPCE_35_)-ct**	70	69	63	30	0.98	49,300	2.2

**Table 3:** Thermal characterization data of PBCE homopolymer and P(BCE_m_BEPCE_n_) and P(BCE_80_NCE_20_) random copolymers.

Polymer	*T*_5%_ °C	*T*_max_ °C	I SCAN						II SCAN					
			*T*_g_ °C	Δ*c*_p_ J/g·°C	*T*_I_ °C	Δ*H*_I_ J/g	*T*_II_ °C	Δ*H*_II_ J/g	*T*_g_ °C	Δ*c*_p_ J/g·°C	*T*_cc_ °C	Δ*H*_cc_ J/g	*T*_II_ °C	Δ*H*_II_ J/g
**PBCE**	381	426	10	0.099	51	1	166	30	8	0.120	-	-	167	30
**P(BCE_85_BEPCE_15_)**	389	428	7	0.128	49	2	140	23	6	0.258	40	19	135	21
**P(BCE_65_BEPCE_35_)**	393	432	10	0.171	50	12	85	6	8	0.210	-	-	-	-
**P(BCE_50_BEPCE_50_)**	400	437	10	0.332	-	-	-	-	11	0.328	-	-	-	-
**P(BCE_80_NCE_20_)**	390	428	15	0.246	49	2	130	19	11	0.298	51	17	130	19
**P(BCE_65_BEPCE_35_)-ct**	394	433	0.3	0.268	50	13	-	-	0.5	0.363	-	-	52	0

**Table 4:** Mechanical characterization and gas transmission rate (GTR) data for PBCE, P(BCE_m_BEPCE_n_), and P(BCE_80_NCE_20_) random copolymers. GTR data are normalized for the thickness of the film sample and are measured at 23 °C, with N_2_, O_2_, and CO_2_ dry gas test.

Campione	*E* (MPa)	σ_b_ (MPa)	ε_b_ (%)	GTR CO_2_ cm^3^cm/m^2^ d atm	GTR O_2_ cm^3^cm/m^2^ d atm	GTR N_2_ cm^3^cm/m^2^ d atm
**PBCE**	830 ± 18	40 ± 2	19.0 ± 4	4.17239	1.29117	0.36585
**P(BCE_85_BEPCE_15_)**	437 ± 22	16 ± 1	424 ± 70	6.05463	2.05468	0.5544
**P(BCE_65_BEPCE_35_)**	82 ± 21	22 ± 4	1198 ± 176	0.01317	0.00517	0.00588
**P(BCE_50_BEPCE_50_)**	6 ± 1	4.0 ± 0.4	1290 ± 205	0.01722	0.00486	0.00705
**P(BCE_80_NCE_20_)**	494 ± 46	14 ± 3	223 ± 77	3.61667	0.88803	0.35319
**P(BCE_65_BEPCE_35_)-ct**	38 ± 3	6.6 ± 0.5	1131 ± 35	15.0923	7.53698	1.80443
